# Designing Lead Optimisation of MMP-12 Inhibitors

**DOI:** 10.1155/2014/258627

**Published:** 2014-01-12

**Authors:** Matteo Borrotti, Davide De March, Debora Slanzi, Irene Poli

**Affiliations:** ^1^European Centre for Living Technology, 30124 Venice, Italy; ^2^Department of Environmental Sciences, Informatics and Statistics, Ca' Foscari University of Venice, 30123 Venice, Italy

## Abstract

The design of new molecules with desired properties is in general a very difficult problem, involving heavy experimentation with high investment of resources and possible negative impact on the environment. The standard approach consists of iteration among formulation, synthesis, and testing cycles, which is a very long and laborious process. In this paper we address the so-called lead optimisation process by developing a new strategy to design experiments and modelling data, namely, the evolutionary model-based design for optimisation (EDO). This approach is developed on a very small set of experimental points, which change in relation to the response of the experimentation according to the principle of evolution and insights gained through statistical models. This new procedure is validated on a data set provided as test environment by Pickett et al. (2011), and the results are analysed and compared to the genetic algorithm optimisation (GAO) as a benchmark. The very good performance of the EDO approach is shown in its capacity to uncover the optimum value using a very limited set of experimental points, avoiding unnecessary experimentation.

## 1. Introduction

Designing molecules with particular properties is usually a long and complex process, in which the nonlinearity of the model, the high number of variables with a leading role, and the categorical structure of these variables can make difficult modelling, experimentation, and analysis. In new drug discovery, a key phase concerns the generation of small molecules modulators of protein function, under the hypothesis that this activity can affect a particular disease state. Current practices rely on the screening of vast libraries of small molecules (often 1-2 million molecules) in order to identify a molecule that specifically inhibits or activates the protein function, commonly known as the lead molecule. The lead molecule interacts with the required target, but it generally lacks the other attributes needed for a drug candidate such as absorption, distribution, metabolism, and excretion (ADME). In order to achieve these attributes, retaining the interaction capacity with the target protein, the lead molecule must be modified. This transformation of the lead molecule is known as lead optimisation. Lead optimisation research involves long synthesis and testing cycles, analyses of the structure-activity relationships (SAR), and quantitative structure activity relationships (QSAR), which are currently the bottleneck of this process [1]. Under traditional approaches these analyses are conducted by experimentation involving an extremely large number of experimental units, which requires large investments of resources and time to reach the target and measure the possible impact on the environment. Computational approaches for the SAR and QSAR analyses, mostly based upon machine learning techniques, have been proposed over the last few years [2–5]. Search and optimisation algorithms inspired by evolution have been also developed and applied with success to drug discovery process and related activities. In Clark [6], different evolutionary algorithms are presented and discussed, such as genetic algorithms (GAs), evolutionary programming (EPs), and evolution strategies (ESs). In this work, several applications of these computational methods have been derived for a wide range of research. Other bio-inspired algorithms such as Ant Colony Optimisation (ACO) and Artificial Neural Networks (ANNs) have been applied in drug discovery [7]. The evolutionary principle is the basic structure of a new approach proposed for designing experiments in an efficient way [8–12]. In this paper we would like to contribute to the development of this research by proposing a new procedure with the objective of finding the optimal value of MMP-12 conducting a very small number of tests and thus with small investments of resources and limited negative impact on the environment. This new procedure is an evolutionary model-based design of experiments: the search for the optimum value is restricted to relatively few experimental points, chosen with the evolutionary paradigm and the information provided by statistical models. Starting from lead molecules, randomization augmented by expert knowledge is used to choose the initial set of compositions to be tested in the laboratory. After chemical synthesis and in vitro screening of these molecules, the resulting response data are evaluated with respect to their capacity to reach the target. They are then transformed according to the operators involved in the evolutionary search and to the information from statistical models estimated on the data. Successive populations of molecules are analysed, modelled, and transformed to generate compositions that are closer to the optimum value. The procedure will be developed and validated, and its efficiency will be measured by a suitable index. Given the successful performance of the simple genetic algorithm for lead optimisation of MMP-12 inhibitors developed by Pickett et al. [1], we will compare our procedure with GAO on this problem. To allow the comparison we will consider the same number of experimental points considered in Pickett et al. [1]. Results exhibit a better performance of our approach in reaching the optimum value and reducing the number of experiments. The paper is organised as follows: in Section 2 we describe the data set on which we developed the procedure and the key idea of the proposed design. In Section 3 we present the results and make comparison with the GAO approach.  Section 4 offers some concluding remarks.

## 2. Materials and Methods

### 2.1. Data Set

**Figure 1:**
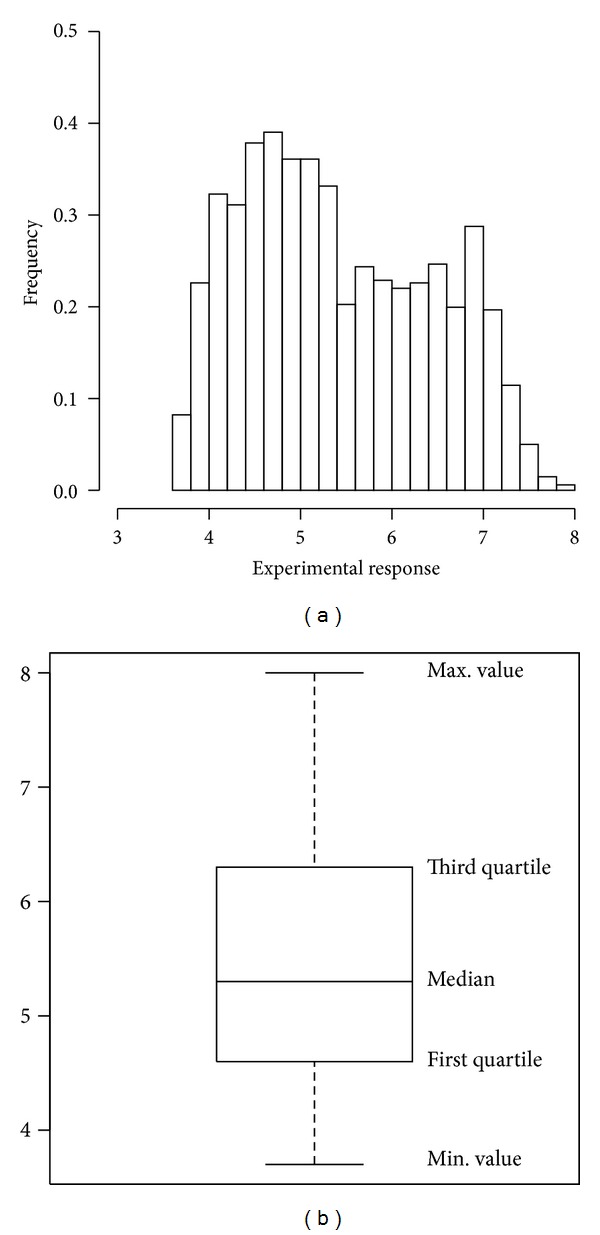
(a) Histogram and (b) boxplot of the experimental response variable.

We build the design for optimisation using a data set presented and analysed by Pickett et al. [1]. These data, available at http://pubs.acs.org, have been constructed by the authors as a test environment on which assessing the effectiveness and the efficiency of new designs for lead optimisations. The data concern a library of 2500 molecules, identified by their chemical compositions (*reagents*) and their experimental response (*activity*). These data represent the whole experimental space. Each data point, coding a particular experiment, is described by two categorical variables, which represent the reagents, each of which can assume 50 different values. The response variable measures the molecular activity of the reaction product. The aim of the analysis is to find the reaction whose product maximises the molecular activity. As a first exploratory analysis of these data we compute a set of descriptive statistics to get some insights into the frequency distribution of the response variable. These data are represented in Figure 1, reporting the histogram and the boxplot. From the exploratory analysis we learn that the maximum value of the molecular activity is 8.00 and the minimum value is 3.40. The mean of the response is equal to 5.28 and the median 5.20; the first quartile is 4.40 and the third quartile is 6.20. These values indicate a right-skewed distribution, and this particular shape is clearly shown in the histogram. In designing experiments for optimisation we will have the objective to find the experimental point with the maximum value of 8.00.

**Figure 2:**
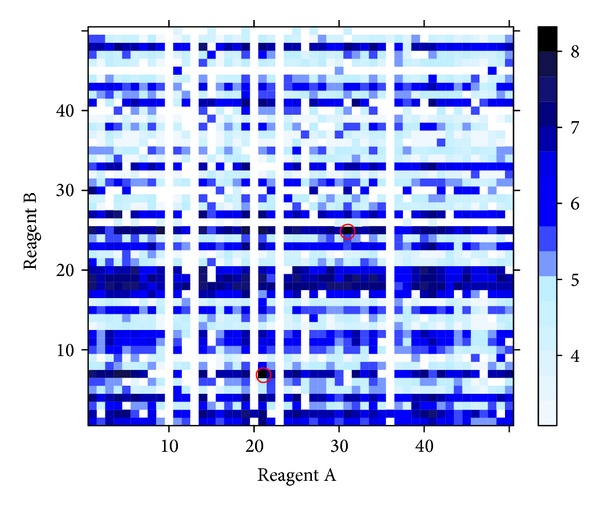
Heatmap for the whole experimental space: 2500 molecules evaluated with respect to their activity. Each square represents a molecule and the colour describes the intensity of the activity, from the light blue to the dark blue. Red circles mark the optimal molecules. White squares represent molecules with not available response.

To describe the behaviour of the response in relation to the molecule composition, we built a *heatmap plot* as presented in Figure 2. The two reagents, say reagents A and B, are reported in the axes of the plot with 50 levels each. For each combination of the reagent levels we can read the value of the molecular activity: high activity values are represented by dark blue squares and small activity values by light blue squares. White squares indicate molecules for which the response is not available. From Figure 2 we can see that just two molecule compositions reach the maximum value of 8.00 (A21; B07 and A31; B25, marked by red circle); also we notice that some reagents, B16 and B20, can give rise to molecules with very high activity values.

### 2.2. Design for Optimisation

An *optimisation* problem is commonly described as follows.

Let *S* be a subset of the Euclidean space ℝ^*d*^ and let *f* be the function *f* : *S* → ℝ^+^. Let **x** = (*x*_1_,…, *x*_*d*_) be a point in *S*; **x** can affect the response variable *y*, and the response can then be described as *y* = *f*(*x*_1_,…, *x*_*d*_). The optimisation problem consists in searching the element x* in *S* such that *f*(x*) ≥ *f*(**x**) for all **x** in *S*.

For the lead optimisation problem that we are addressing in this research the dimension *d* equals 2, since two variables are considered in the dependence relation.

These variables are categorical variables, namely, the reagents, and can take *l* = 50 different levels.

With this setting of the problem the experiment is run to provide each experimental point with a response value that is a measure of the activity of the resulting molecule. The experimental data set is then (**X**; **y**), with **X** being an (*N* × 2)-matrix, where *N* = 2500 is the size of *S*, and **y** being an *N*-vector. This data set represents the evidence for inferring the dependence relation among variables and identifies the design point that gives the optimum value of the response.

The *design* problem for optimisation consists in finding a small set of experimental points that contain the relevant information to reach the optimal value. Our contribution to address this problem is to adopt evolution as a paradigm to build a design approach guiding the evolution with the information achieved by statistical models.

### 2.3. Evolutionary Design for Optimisation

Building on the evolutionary strategy we introduce a new approach, named evolutionary design for optimisation (EDO), testing a very small set of different experimental points able to find the optimal response value or the region of optimality. This approach evolves an initial design through *K* generations by means of a set of genetic operators (selection, recombination, and mutation) that are built on the information provided by models estimated on the data of each design generation (**X**_**k**_; **y**_**k**_), *k* = 1,…, *K*.

For the lead optimisation problem addressed in this research we build the EDO approach with the objective of achieving the optimum value of the molecule activity testing 140 experimental points as in genetic algorithm optimisation (GAO) strategy introduced by [1]. More specifically, we select an initial population of experimental points **X**_1_, consisting of two sets of compounds: the first set is created by assigning at each level of reagent A a randomly selected level of reagent B; then, the second set is created by assigning at each level of reagent B a randomly selected level of reagent A. Each of these sets include 50 compounds, and then the design consists of *m*_1_ = 100 different compounds. Each of these experimental points receives a response value **y**_1_. Therefore on the data set (**X**_1_; **y**_1_), a statistical model is estimated to achieve information on the goodness of these compositions in reaching the target of the optimisation. In this research we developed several Monte Carlo simulation studies comparing different classes of statistical models in their predictive capacity, and we selected the random forest model [13–15] as our best choice. The random forests are regression methods frequently used when the relationship between response and predictors is complex, and the predictors are categorical variables [16].

Following the evolutionary paradigm, we then adopt a selection operator where the probability of each experimental point to be selected for next generation is proportional to the square of the response value, according to the following expression:

(1) $$\begin{array}{c} \pi_{i}=\frac{y_{i}^{2}}{\sum_{i=1}^{n}y_{i}^{2}}, \end{array}$$

where *n* is the total number of experimental points tested up to and including the current generation. For this optimisation problem we select 10 compounds, and each selected compound is then recombined in order to create a set of new and not already tested points. We estimate a random forest model and proceed in the following way: for each selected compound we fix the reagent, randomly chosen between A and B, and its corresponding level in the compound, and then we generate all the possible compounds by changing the 50 levels of the other reagent. For all these new generated experimental points we then predict the responses using the estimated random forest model and the compound with the highest estimated response value is considered for the following generation. A mutation operator is then performed (with probability *P* = 0.05) by randomly changing one reagent level. The second population of *m*_2_ = 10 experimental points **X**_2_ is then defined. We iterate the procedure across generations until the optimum value is archived or a stopping rule is satisfied.

**Figure 3:**
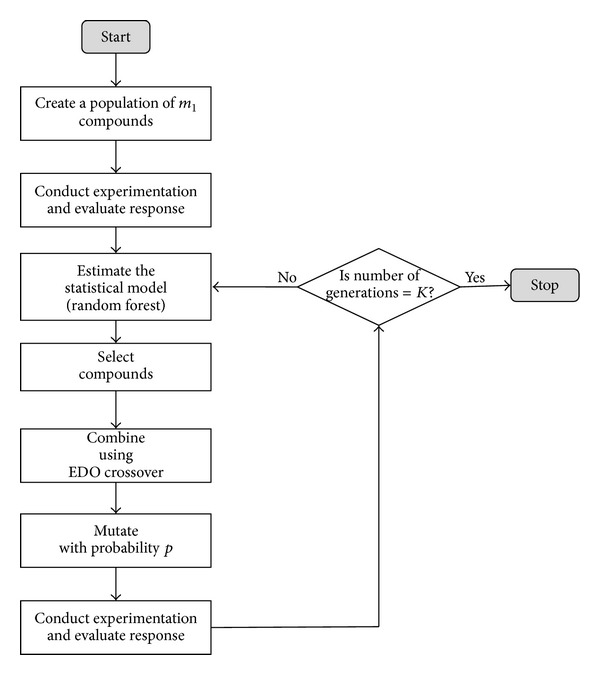
Flow diagram of EDO design for lead optimisation.

The EDO procedure is represented in Figure 3 and described as follows.

1. Create a population of *m*_1_ compounds (*m*_1_ = 100).
2. Conduct experimentation and evaluate the response.
3. Estimate the statistical model (i.e., random forest).
4. Select a compound (according to (1)).
5. Combine the reagents using EDO crossover as follows:
  - select a reagent in a random way;
  - generate all the possible compounds by changing the 50 levels of the other reagent;
  - infer the molecules activity with the estimated model (random forest);
  - select the compound with the highest estimated activity value for next generation.
6. Repeat step's 4 and 5 until the 10 new compounds are created.
7. Mutate the new compounds with *p* = 0.05.
8. Conduct experimentation and evaluate the response.
9. If number of generations is equal to *K* stop the algorithm. Otherwise repeat steps from 3 to 8.

The EDO procedure is developed in R code (http://cran.r-project.org/) and uses randomForest package [16]. Random forest model is estimated running 500 trees, and model selection is performed with standard parameterisation of the package.

### 2.4. Measure of the Design Goodness

To evaluate the design goodness we introduce two criteria. The first criterion is a measure of the distance between the response value of the best experimental point provided by the design and the actual optimal value of the whole system response. In particular, let ${\hat{y}}_{\max}$ be the maximum value found by the design, and let *y*_max_ and *y*_min_ be the known maximum and minimum of *y* on the whole search space. The design goodness for optimisation criterion (DGO) is

(2) $$\begin{array}{c} \text{DGO}=1-\frac{\left|{\hat{y}}_{\max}-y_{\max}\right|}{\left|y_{\min}-y_{\max}\right|}. \end{array}$$

This measure ranges in value from 0 to 1. The second criterion of design goodness evaluates the capacity of the approach to find response values in defined regions of optimality. We derive this indicator by counting the number of experimental points with response value greater than a defined threshold. This threshold is identified by the right tail area of the response values distribution measured by the probability values *α* = 0.01 and *α* = 0.05. The DGO_*α*_ can be expressed as follows:

(3) $$\begin{array}{c} \text{DG}{\text{O}}_{\alpha }=\frac{\sum_{i=1}^{m}I\left(y_{i}\geq y_{\alpha }\right)}{\sum_{j=1}^{N}I\left(y_{j}\geq y_{\alpha }\right)}, \end{array}$$

where *m* is the total number of tested compounds, *N* is the number of compounds of the whole experimental space, *y*_*α*_ is the percentile of the response distribution at *α* level, and *I*(·) is the indicator function. The DGO_*α*_ ranges from 0 to 1, where DGO_*α*_ = 0 indicates that no compound selected by the design is in the optimal region and DGO_*α*_ = 1 indicates that all the selected compounds are in the optimal region.

## 3. Results

**Figure 4:**
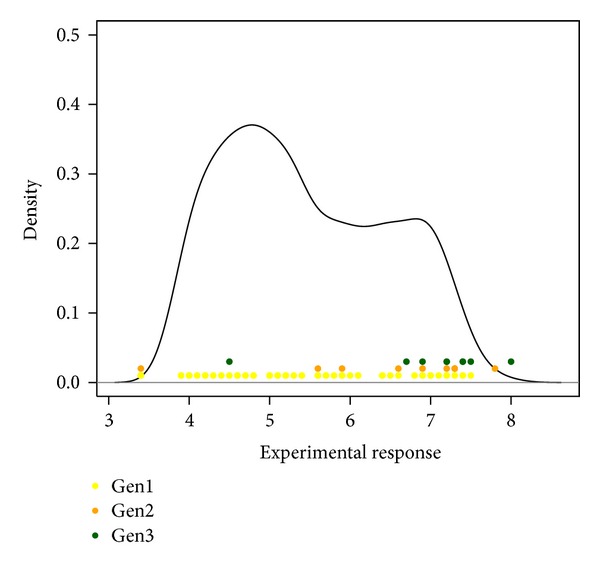
Estimated density function of response variable of the whole experimental space (2500 compounds). In yellow the response values of the initial population composed of *m*_1_ = 100 compounds. The next populations, composed of *m*_*k*_ = 10 compounds, *k* = 2,3, are represented by orange and green points.

To derive an efficient design for the lead optimisation problem we apply the EDO design and search in the experimental space of 2500 compounds for the optimum value. The initial population of *m*_1_ = 100 compounds sampled from the whole experimental space (as in Section 2.3) are spread in the response distribution as described in Figure 4.

**Figure 5:**
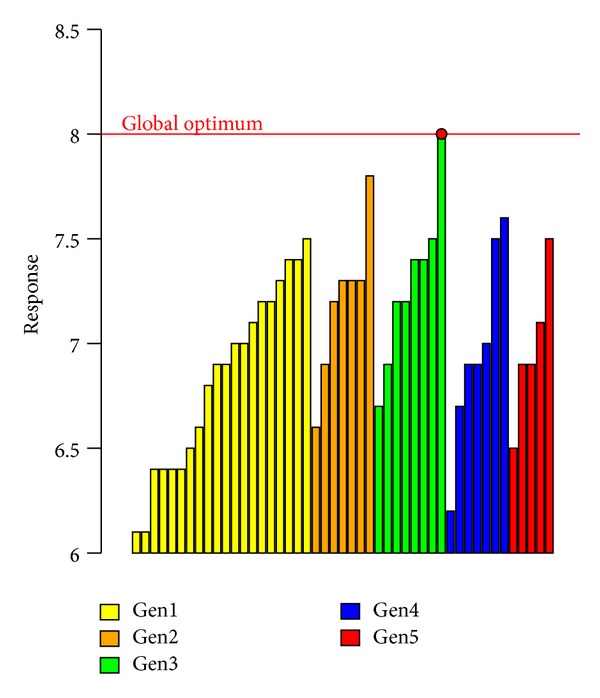
EDO response values greater than the threshold equal to 6, ordered by generation.

This result and the evolution of the experimental response achieved by generations are shown in Figure 5 (response values greater than 6) where we notice that EDO finds the optimal value of 8.00 (global optimum of the whole experimental space) at the third generation. We also notice that this approach is able to find a set of very good values close to the optimum.

**Figure 6:**
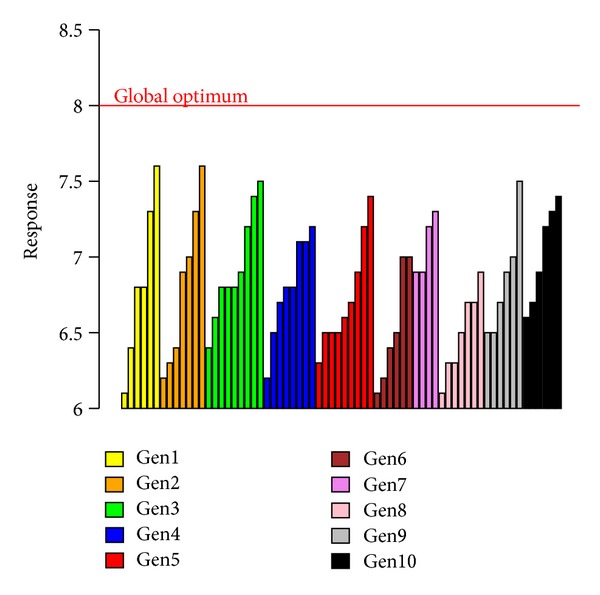
GAO response values greater than the threshold equal to 6, ordered by generation.

To evaluate the performance of the procedure we developed a comparison of the EDO approach with the GAO, which is considered as a benchmark for this new approach. Reporting the response values by generations of the GAO in Figure 6 (response values greater than 6) we observe that the simple GA, without the statistical modelling contribution, is not able to find the optimum value testing 140 compounds and conducting 10 generations of experiments. Moreover we notice that most of the GAO response values remain under the threshold of 7.50.

Since the GAO approach has shown very good performance with respect to the traditional approach in lead optimisation, the results achieved under EDO design can be regarded as satisfactory. The EDO design on this set of data discovers the global optimum testing just 120 compounds.

Computing the design goodness criterion presented in (2) we compare the optimal response values achieved by the GAO, ${\;}_{\text{GAO}}{\hat{y}}_{\max}=7.60$, and by EDO, ${\;}_{\text{EDO}}{\hat{y}}_{\max}=8.00$, with the known optimum value of the whole space and derive the following measures of design goodness:

(4) GAODGO=0.90,  EDODGO=1.00,

confirming the superior performance of the EDO design.

As a principal result the new procedure has been able to discover this value testing just 120 compounds and conducting 3 generations of the algorithm. Furthermore, the comparison in performance between EDO and GAO can be realised considering a region of optimality instead of the single optimal value.

Deriving the measure DGO_*α*_, as presented in (3) for both optimisation approaches, we obtain that, in the right tail region of the distribution of the responses with *α* = 0.01, the EDO approach can find 29% of the best compounds, while the GAO is able to discover just 17% of these compounds. We achieve similar results considering the region of the right tail distribution *α* = 0.05, where EDO approach outperforms the results of GAO finding 21% of the best experimental units. These results are reported in Table 1.

We test the statistical significance of the null hypothesis that EDO and GAO have equal proportion of responses in the best response region. From this statistical test, we compute the one-tailed *P* value to evaluate the improvement of EDO compared to GAO: we obtain *P* value = 0.0991 considering the optimality region *α* = 0.01 and *P* value = 0.1361 considering the optimality region *α* = 0.05. These statistical tests confirm the improvement of EDO design compared to GAO design.

**Figure 7:**
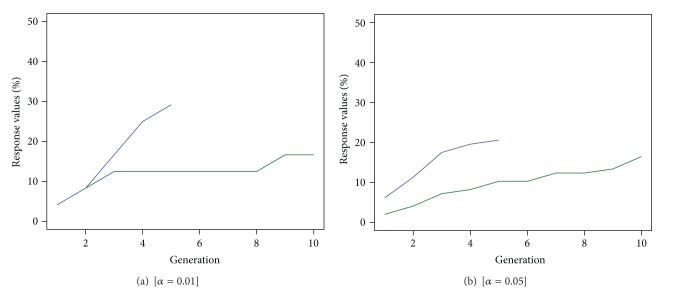
Evolution of the proportion of the best compounds found by the optimisation designs in the *α* = 0.01 optimality region (a) and *α* = 0.05 optimality region (b). The EDO design is represented by the blue line and the GAO by the green line.

**Figure 8:**
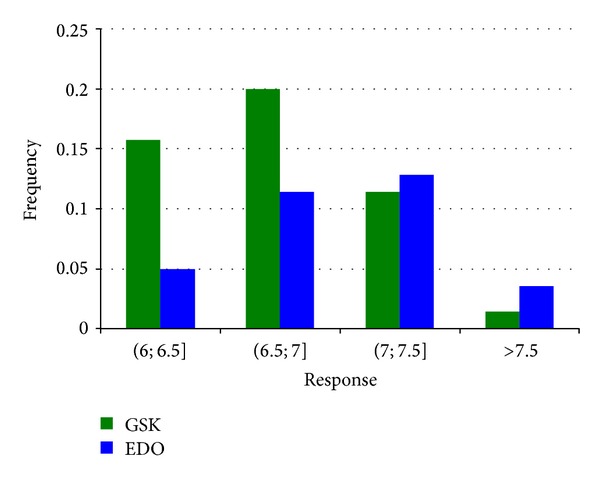
Frequency distribution of the response for values greater than the threshold equal to 6. EDO compounds are represented in blue, and GAO compound, are represented in green.

Studying the evolution of the proportion of the best compounds found in the optimality area and described in Figure 7, we notice that this proportion increases much faster and more intensively for the EDO design than for the GAO design. Selecting the region of optimality *α* = 0.01 (Figure 7(a)) the number of responses from the EDO design that fall in this area increases rapidly reaching in 5 generations 29% of the best responses, instead of the 17% of the GAO design. Similar behaviour can be observed for the region of optimality with size *α* = 0.05 (Figure 7(b)). Finally we derived the frequency distributions of the best response values (*y*_*i*_ > 6.0) comparing the EDO and GAO optimisation procedures, as described in Figure 8. We can observe that the proportion of the compounds achieved with EDO design (blue bars) grows for increasing values of the responses. Moreover for values greater than 7.5 this proportion is very high and is much higher than the proportion achieved with GAO procedure.

In order to study the robustness of EDO design with respect to changes in the initial population, we performed a simulative study where we run our algorithm 100 times with different initial populations.

As a result, 90% of the simulations have been able to find greater or equal response values with respect to the best result obtained in Pickett et al. [1]. This result shows the robustness of the EDO with respect to the choice of the initial population confirming the good performance of the approach.

## 4. Concluding Remarks

In this research we addressed the lead optimisation problem for drug discovery process by developing a design for experiments which is evolutionary and based on the information provided by statistical models. The motivation of this research is to give a contribution to the study of finding an efficient design that tests a very small set of experimental points instead of the whole space, which due to the high dimensionality of the system or the high number of the variable levels may be very large.

The approach that we derived outperforms the GAO methodology developed by Pickett et al. [1]. In fact selecting 120 experimental points from the whole search space, EDO is able to find the global optimum value. These results suggest that the development of an evolutionary design as in GAO is certainly successful in optimisation problems, but the introduction of statistical models at each step of the evolution as in EDO can improve the optimisation procedure.

## Figures and Tables

**Table 1:** Evaluation of the goodness of the design in terms of optimality area: the proportion of the best compounds found by EDO and GAO procedures over the given thresholds.

	Proportion of exp. points with responses in the optimality region (%)	
	EDO	GAO
DGO_0.01_	29	17
DGO_0.05_	21	16
